# Supplementary material for: Ualign: pushing the limit of template-free retrosynthesis prediction with unsupervised SMILES alignment
Source: J Cheminform. 2024 Jul 15;16:80. doi: 10.1186/s13321-024-00877-2 (PMC11247856; doi:10.1186/s13321-024-00877-2)
Supplement: Supplementary file 1 [file 13321_2024_877_MOESM1_ESM.pdf]

Supplementary material of

UAlign: Pushing the Limit of Template-free Retrosynthesis Prediction with Unsupervised SMILES Alignment

Kaipeng Zeng[1], Bo Yang[2], Xin Zhao[1], Yu Zhang[1], Fan Nie[3], Xiaokang Yang[1], Yaohui Jin[1*], Yanyan Xu[1*]

[1*]MoE Key Laboratory of Artificial Intelligence, AI Institute, Shanghai Jiao Tong University, Shanghai, 200240, Shanghai, China.
[2]Frontiers Science Center for Transformative Molecules (FSCTM), Zhangjiang Institute for Advanced Study, Shanghai Jiao Tong University, Shanghai, 200240, Shanghai, China.
[3]Department of Computer Science and Engineering, Shanghai Jiao Tong University, Shanghai, 200240, Shanghai, China.

*Corresponding author(s). E-mail(s): jinyh@sjtu.edu.cn; yanyanxu@sjtu.edu.cn;

1 Notations

Supplementary Table 1 lists the notations used to facilitate reading.

2 Dataset

The open benchmark datasets are presented as follows.

USPTO-50K. The USPTO-50K is of high quality, we only remove the reactions whose products only contain a single atom from the dataset.

USPTO-FULL. The raw data of USPTO-FULL provided by Dai et al has error annotations and other problems. Thus we perform the following steps to clean up the data.

Supplementary Table 1: Notations for facilitating reading.

Notation	Description
$G = (V, E)$	a graph with nodes V and edges E
$rank(a, O)$	the position of atom a in DFS order O
$root(G, O)$	the atom (node) of G with the minal rank in O
$Smiles(G, O)$	The SMILES of molecule graph G generated via DFS order O
$M = (V_M, E_M)$	a molecule graphs
$M' = (V_{M'}, E_{M'})$	a molecule graph
$\tilde{M} = (V_M \cup V_{M'}, E_M \cup E_{M'})$	a molecule graph consist of two isolated molecules M and M'
O_M	a DFS order of molecule graph M
$O_{\tilde{M}}$	a DFS order for molecule graph $\tilde{M}$
$\mathcal{R}$	the set of all reactant molecule graphs
$P = (V_P, E_P)$	the product molecule graph
$R = (V_R, E_R)$	a single reactant molecule graph
$\mathcal{D}(V_P), \mathcal{D}(V_R)$	the set of all possible DFS order for product molecule graph / a single reactant molecule graph
O_P, O_R	a DFS order of product, a DFS order of reactant molecule graphs
$CO(R, O_P)$	the DFS order of reactant R that has a nearly consistent atomic appearance sequence with O_P
$OPSmiles(\mathcal{R}, O_P)$	the order-preserving reactants SMILES of $\mathcal{R}$ based on DFS order O_P
$h_u^{(k)}$	the node feature of node u at k-th iteration of message passing.
$e_{u,v}^{(k)}$	the edge feature of edge between node u and v at k-th iteration of message passing.
H	the output of the encoder, i.e. the encoded node features
Z	the input token embedding for Transformer decoder
$\hat{H}$	the order-aware node features
$\hat{Z}$	the output token embedding of Transformer decoder
$\hat{T}$	the predicted token logits list
$\hat{t}_i$	the i-th token logits in the predicted token list
T	the ground truth token list
t_i	the i-th ground truth token

- remove the reactions where different atoms share the same atom map number.
- remove the reactions whose product is consist of a single atom.
- remove the reactions with invalid SMILES. The empty SMILES is considered as invalid SMILES too.
- remove the reactions where product contains atoms that do not appear in reactants.
- remove the reactants that all atoms do not appear in the product.

USPTO-MIT. The raw data of USPTO-MIT provided by [4]. The original dataset contains reactions that have multiple product molecules. And the reagents are put together with reactions. Thus we perform the following steps to make the dataset more suitable for retrosynthesis prediction task.

- remove the reactions whose product is consist of a single atom
- remove the reagents from the reactants. We consider the molecules whose atoms have no intersection with the product as reagents.
- remove the reactions with multiple product molecules.

The statistical information of the datasets used in this work is summarized in Supplementary Table 2.

Supplementary Table 2: **Summary of datasets used in this paper.** #Train/#Valid/#Test denotes the number of samples in the training/validation/test set, respectively. #Total is the sum of #Train, #Valid and #Test.

Dataset	#Train	#Valid	#Test	#Total
**USPTO-50K**	40,006	5,001	5,007	50,016
**USPTO-MIT**	395,498	29,076	38,648	453,222
**USPTO-FULL**	768,679	96,076	96,015	960,770

3 Evaluation Metrics

Top-k Accuracy. We use the conventional top-k accuracy to evaluate the performance of model. A prediction result is considered as correct if and only if all the reactants are correctly predicted.

Top-k SMILES Validity. As SMILES is considered as correct when it can be identified by RDKit [6]. The top-k SMILES validity is calculated as $\frac{1}{N\times k}\sum_{i=1}^{N}\sum_{j=1}^{k}\mathbb{1}(\text{SMILES is valid})$, where N is the number of evaluated samples.

Top-k round-trip Accuracy. There may be multiple methods to synthesize a given product. Evaluating the model's performance solely based on top-k accuracy may lead to biased results. Therefore, in addition to top-k accuracy, we also employ top-k round-trip accuracy as an extra metric to evaluate the model's performance. Top-k round-trip accuracy measures the percentage of predicted reactants that can undergo a reaction and yield the given product. To align with our baseline, we use the

Molecule Transformer [8] as the forward reaction prediction model. The calculation of top-k round-trip accuracy follows the same approach as described in [10].

4 Visualization of the Cross-Attention Coefficients

We visualize the cross-attention maps across different transformer decoder layers using the checkpoint trained on the USPTO-50K dataset. The outcomes are depicted in Supplementary Fig. 1.

5 Implementation details

5.1 Generation of Order-Preserving Reactant SMILES

Given a traversal order O_P of product P, there might be multiple DFS orders of a reactant molecule that satisfy the condition to be the O_P-corresponding order. This is because reactants might contain atoms and substructures that are not present in the final product. Also, directly solving this problems is very difficult. Thus, we use an approximate algorithm, which is shown in Supplementary Algorithm 1 to generate the corresponding order defined in Eq. 2 given product molecule P, reactant molecule R and a DFS order O_P of P, where $am(i)$ represents the atom map number of atom i.

5.2 Model Implementation details (Training and Inference)

We implement our model based on `Pytorch 1.13` [7] and `torch_geometric 2.2.0` [2]. For model for USPTO-50K, we set the hidden size as 512, encoder layer as 8, decoder layer as 8 and the number of attention heads as 8. For model for USPTO-FULL, we set the hidden size as 768, encoder layer as 8, decoder layer as 8 and the number of attention heads as 12. The dropout ratio for both model is set as 0.1. The highest learning rate of each model as set as 1.25e-4. We slowly increase our learning rate to the highest in the first few epochs and slowly decrease it using exponential decay. The model is trained with Adam optimizer [5]. The maximum number of training epochs for both stages is established at 300, and an early stopping strategy is implemented, which terminates the training process if the model fails to demonstrate performance improvement on the validation set for ten consecutive epochs.

It is important to note that a prescribed DFS order may implicitly capture the distribution of reactant sub-groups within the product, which could inadvertently result in information leakage. To mitigate this issue, we employ a DFS order during the inference process that generates a canonical SMILES representation for the product molecule, ensuring a standardized input that precludes information leakage.

6 Initial Node Features and Edge Features

We use the atom and bond descriptors provided by `Open Graph Benchmark` [3]. There are nine atom descriptors including atomic number, chirality, formal charge and other properties that can be calculated by RDKit [6]. And three bond descriptors are used, containing bond type, bond stereochemistry as well as whether the bond is conjugated.

Supplementary Algorithm 1 Get Corresponding Order

Input: product graph $P = (V_P, E_P)$, DFS order of product O_P, reactant $R = (V_R, E_R)$.

function GET_RANK(O_P: DFS order of P, x: atom, R: reactant, $AMRank$: a dict, Vis: a set)

  Add x into Vis.

  Initialize min_son as ∞.

  for $y \in$ GET_NEIGHBOR(x, R) **do**

    if $y \notin Vis$ **then**

      GET_RANK(O_P, y, R, $AMRank$, Vis).

      $min\_son = \min(min\_son, AMRank[am(y)])$.

    end if

  end for

  if $x \in O_P$ **then**

    $AMRank[am(x)] = rank(x, O_P)$.

  else

    $AMRank[am(x)] = min\_son$.

  end if

end function

function GET_CO(CO: a list, x: atom, R: reactant, $AMRank$: a dict, Vis: a set)

  Add x into Vis.

  Append x to CO.

  Initialize N as an empty list.

  for $y \in$ GET_NEIGHBOR(x, R) **do**

    if $y \notin Vis$ **then**

      Append y to N.

    end if

  end for

  Sort N in ascending order according to $AMRank[am(x)], x \in N$.

  for $y \in N$ **do**

    GET_CO(CO, y, R, $AMRank$, Vis).

  end for

end function

Initialize Vis as an empty set.

Initialize CO as an empty list.

Initialize $AMRank$ as a empty dict.

Set $root$ as the atom $x \in V_R$ that $am(x) = am(root(P, O_P))$.

GET_RANK(O_P, $root$, R, $AMRank$, Vis)

Reset Vis as an empty set.

GET_CO(CO, $root$, R, $AMRank$, Vis).

Output: $CO(R, O_P) = CO$

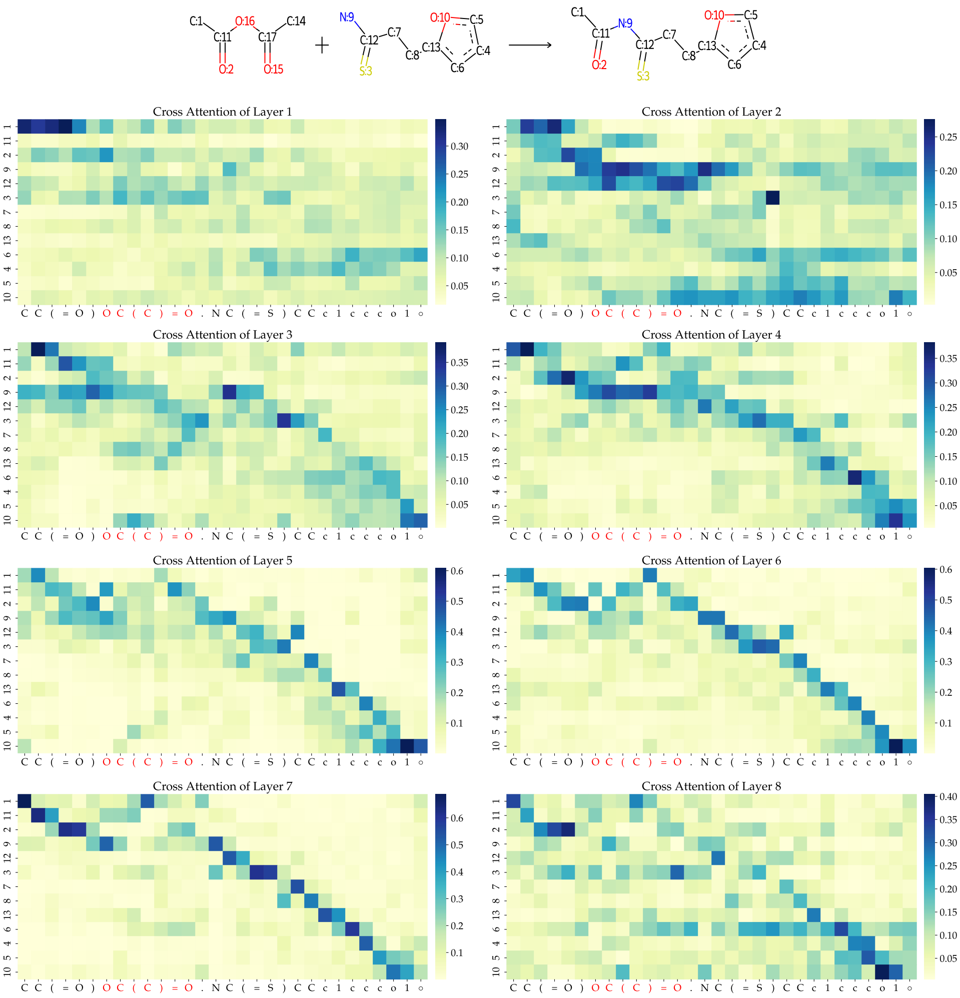

Supplementary Fig 1: Visualization of cross attention over product atoms and predicted tokens of different Transformer decoder layers. The number on the y-axis is the map number of atoms in the product. The reactants atoms that not appear in product is colored red in the x-axis. ∘ represents the end token.

All the descriptors provide integer outputs. Thus we set up learnable embedding tables for each descriptor. The initial node features and edge features are then obtained by summing up the embedding corresponding to the output of each descriptor.

7 Results of Multi-step Retrosynthesis Planning of Baselines

We visualize the results of multi-step retrosynthesis planning for strong SMILES-based baseline Retroformer [10] and graph-based baseline GraphRetro [9], using the case from Sec. 3.5. The results are presented in Supplementary Fig. 2 and Supplementary Fig. 3.

References

[1] Dai H, Li C, Coley C, et al (2019) Retrosynthesis prediction with conditional graph logic network. Advances in Neural Information Processing Systems 32

[2] Fey M, Lenssen JE (2019) Fast graph representation learning with PyTorch Geometric. In: ICLR Workshop on Representation Learning on Graphs and Manifolds

[3] Hu W, Fey M, Zitnik M, et al (2020) Open graph benchmark: Datasets for machine learning on graphs. Advances in neural information processing systems 33:22118–22133

[4] Jin W, Coley C, Barzilay R, et al (2017) Predicting organic reaction outcomes with weisfeiler-lehman network. Advances in neural information processing systems 30

[5] Kingma DP, Ba J (2014) Adam: A method for stochastic optimization. arXiv preprint arXiv:14126980

[6] Landrum G, et al (2013) Rdkit: A software suite for cheminformatics, computational chemistry, and predictive modeling. Greg Landrum 8:31

[7] Paszke A, Gross S, Massa F, et al (2019) Pytorch: An imperative style, high-performance deep learning library. Advances in neural information processing systems 32

[8] Schwaller P, Laino T, Gaudin T, et al (2019) Molecular transformer: a model for uncertainty-calibrated chemical reaction prediction. ACS central science 5(9):1572–1583

[9] Somnath VR, Bunne C, Coley C, et al (2021) Learning graph models for retrosynthesis prediction. Advances in Neural Information Processing Systems 34:9405–9415

[10] Wan Y, Hsieh CY, Liao B, et al (2022) Retroformer: Pushing the limits of end-to-end retrosynthesis transformer. In: International Conference on Machine Learning, PMLR, pp 22475–22490

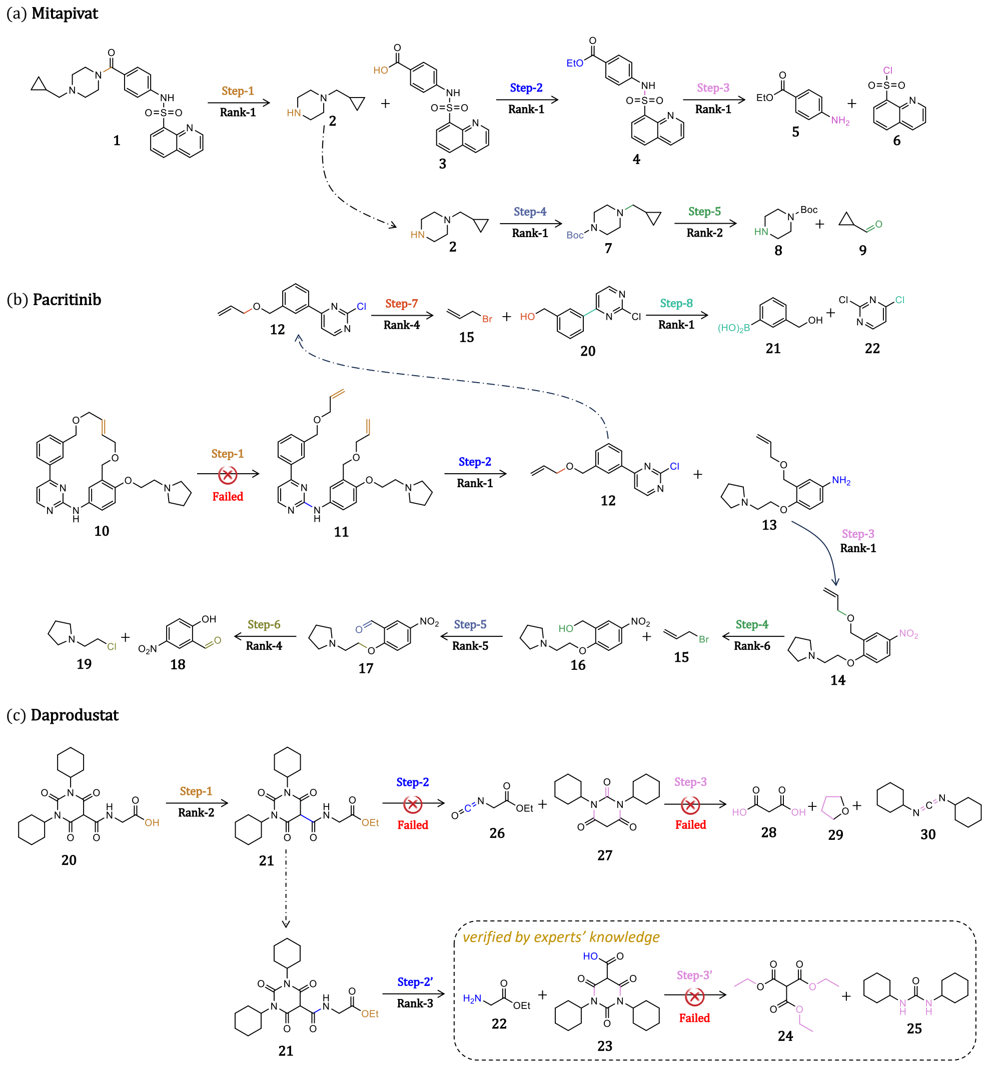

Supplementary Fig 2: Multistep retrosynthesis predictions by Retroformer. (a) Mitapivat (b) Pacritinib (c) Daprodust. The reaction centers and leaving groups are highlighted in different colors. The pathway pf molecules (a) and (b) come from literature. For molecule (c), we provide both the literature-based synthetic pathway and a pathway verified by chemical experts.

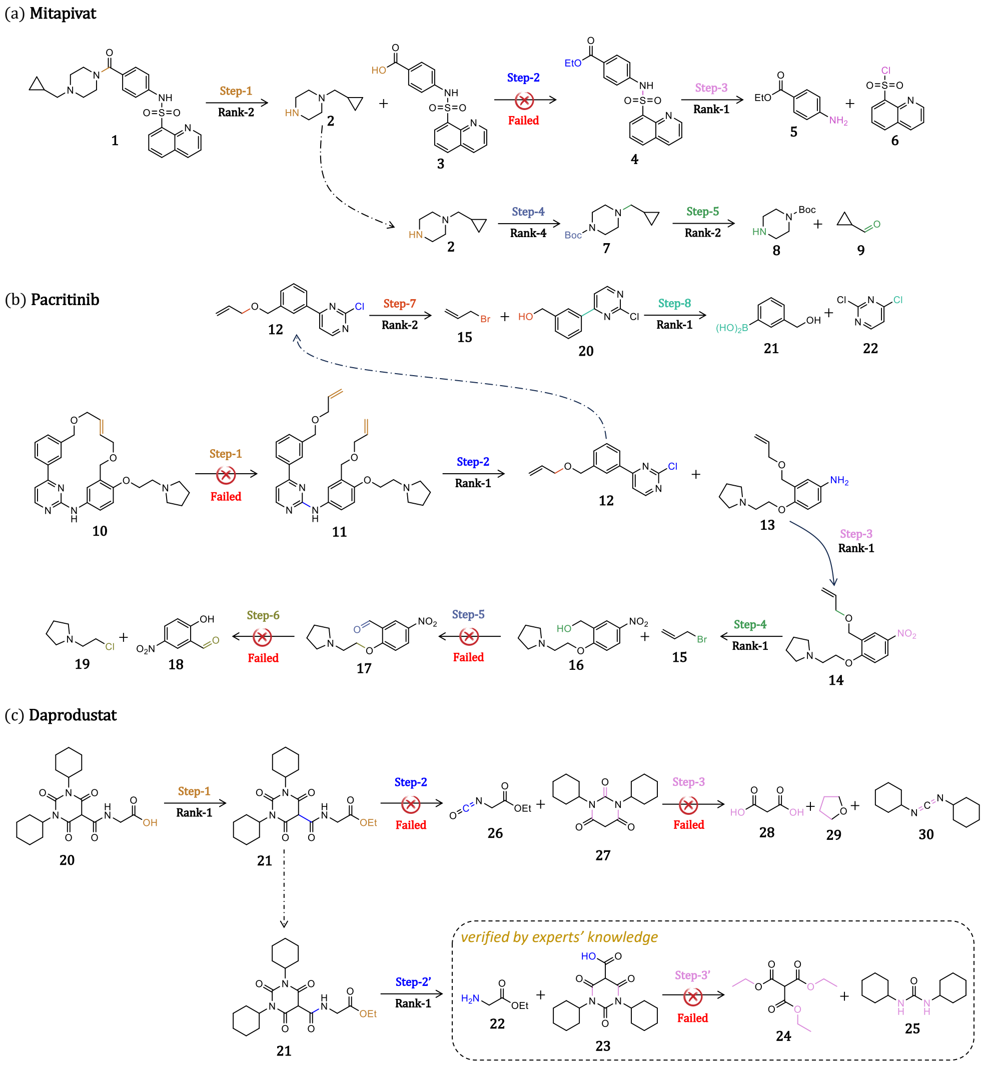

Supplementary Fig 3: Multistep retrosynthesis predictions by GraphRetro. (a) Mitapivat (b) Pacritinib (c) Daprodust. The reaction centers and leaving groups are highlighted in different colors. The pathway pf molecules (a) and (b) come from literature. For molecule (c), we provide both the literature-based synthetic pathway and a pathway verified by chemical experts.
